# Multifocal Langerhans cell sarcoma involving epidermis: a case report and review

**DOI:** 10.1186/1746-1596-7-99

**Published:** 2012-08-14

**Authors:** Changsong Wang, Yanping Chen, Chunfang Gao, Jian Yin, Hong Li

**Affiliations:** 1 Department of Pathology, 150th Hospital of PLA, Luoyang, China; 2 Department of Otorhinolaryngology and Head-Neck Surgery, Xinqiao Hospital, Third Military Medical University, Chongqing, China

**Keywords:** Langerhans cell sarcoma, Multiple focus, Langerin, CD1a

## Abstract

**Objective:**

To study the clinico-pathological characteristics of Langerhans cell sarcoma (LCS) which involving epidermis.

**Methods:**

A case of primary multifocal LCS was analyzed in histopathology and immunophenotype.

**Results:**

A 41-year-old man with multifocal cutaneous LCS involving the inguina and waist was reported. Clinical and pathology data were available. Neoplastic cells with markedly malignant cytological features were observed. Tumor cells exhibited irregular shape with abundant and eosinophilic red staining cytoplasm; large, irregular-shaped, showing lobulated or dented nucleus and some cells with a longitudinal nuclear groove and prominent nucleoli. The tumor cells expressed CD1a, Langerin (CD207), S-100 protein, CD68 and vimentin, and did not express pan-T or B cell markers and epithelial markers. The patient died less than 1 year after diagnosis due to local recurrence and metastasis to the lung, despite the administration of local radiation and chemotherapy.

**Conclusions:**

LCS is a tumor with markedly malignant cytological features that originates from Langerhans cells. Primary multifocal neoplasms involving epidermis is even rare. Accurate diagnosis is based on the histopathological and immunohistochemical of the tumor cells.

**Virtual slide:**

The virtual slide(s) for this article can be found here: http://www.diagnosticpathology.diagnomx.eu/vs/1182345104754765.

## Background

The Langerhans cell tumors were classified into Langerhans cell histiocytosis (LCH) and Langerhans cell sarcoma (LCS) by the World Health Organization (WHO) in 2001. LCS is a rare dendritic cell tumor, and the WHO defined it as having typical features of malignant cytology as a high-grade variant of LCH [1]. The diagnosis of LCS is difficult, and it is necessary to differentiate it from many other tumors, such as metastatic cancer, malignant melanoma, anaplastic large cell lymphoma, myeloid sarcoma, malignant fibrous histiocytoma and LCH. In addition, the tumors of LCH and LCS both arise from dendritic cells and have the same histological features, immunophenotype, and Birbeck granules in the cytoplasm. Therefore, differentiating between LCS and LCH is difficult. LCS occure at any age and involve many organs or tissues, such as bone, lung, lymph nodes, liver and soft tissues. LCS involved epidermis to form multiple focus was still rare, in the present report, we reported such a case of LCS.

### Case presentation

#### Clinical summary

A 41-year-old man with a slowly growing soft tissue mass (which was painful when manual pressure was applied), which developed over a period of 6 months, over the anterior iliac spine was reported. The wound healed poorly after the operation and recurred as a cord-like tissue from the wound to the left groin. This cord-like tissue disappeared upon administration of antibiotic therapy. Then, a small mass with no pain emerged at his left groin, and treatment with antibiotics had no effect. The mass gradually become large and solid. Another mass emerged at the right groin during the first month.

Physical examination indicated that the masses were located at the bilateral groins and at the left waist with a sharp border. The masses at the right groin, left groin and left waist had sizes of 3 cm × 6 cm, 15 cm × 8 cm and 10 cm × 8 cm, respectively. There were no swollen lymph nodes at the surface of the skin.

Upon operating, the mass was determined to be located subcutaneously and had a clear border.

### Pathological findings

**Figure 1:**
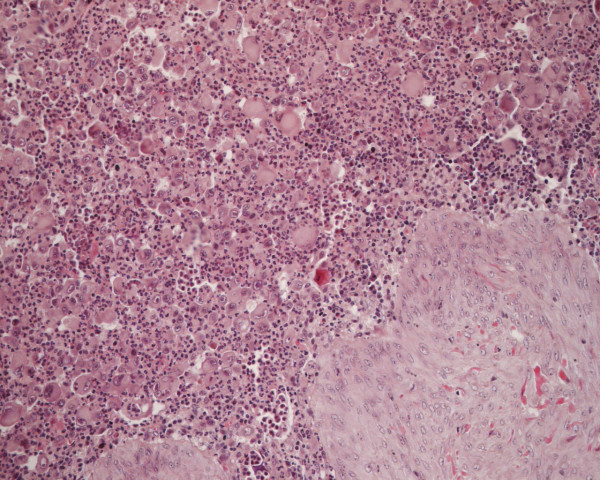
Hematoxylin-eosin staining of LCS (100×).

**Figure 2:**
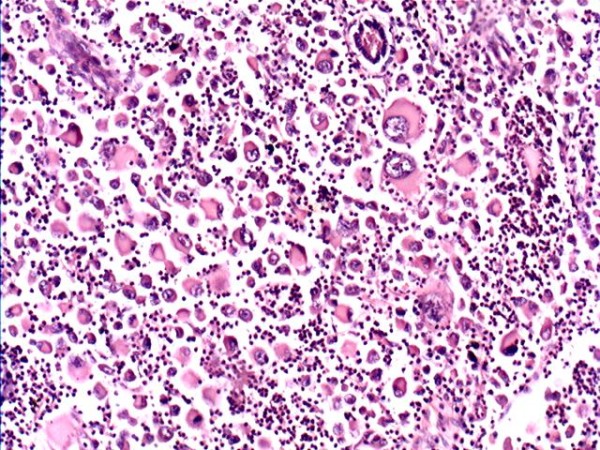
Hematoxylin-eosin staining of LCS (200×).

The specimens that were obtained consisted of a few broken pieces and a little subcutaneous tissue, measuring 13 cm × 10 cm × 4 cm. The cut surface was gray-white, soft and had a pseudo-membrane. Microscopically, the majority of the larger tumor cells were located diffusely under the dermis and subcutaneous tissue (Figure 1). The cell morphology varied as follows: irregular shape with abundant and eosinophilic red staining cytoplasm; large, irregular-shaped, showing lobulated or dented nucleus and some cells with a longitudinal nuclear groove and prominent nucleoli; and a large number of multinuclear giant cells with a few or several dozen nuclei. These cells had significantly malignant cytological features. A high mitotic rate (more than 30 mitoses per 10 high power fields) was observed. The tumor cells were surrounded by a small amount of fibrous tissue. A large number of neutrophils, giant cells, a small number of plasma cells, eosinophils and lymphocytes in phagocytic cells were observed, interspersed with the tumor cells (Figure 2).

### Immunohistochemical staining and results

The formalin-fixed paraffin-embedded tumor samples were sliced in 3-μm-thick sections, and the sections were prepared for hematoxylin and eosin staining and for immunohistochemical detection.

The sections were pressure cooked for 15 min in 10 mM citrate buffer (pH 6.0) for antigen retrieval after being dewaxed with xylene and rehydrated through a graded series of ethanol. The sections were then incubated in phosphate-buffered saline containing horse serum albumin, and the primary antibodies were added for the reaction. The reaction with the primary antibodies was performed at 4°C overnight, then the second antibody was added and incubated at 37°C for 30 min. Finally, the sections were treated with 3% H_2_O_2_ to reduce endogenous peroxidase activity, and the reaction was visualized with DAB.

**Figure 3:**
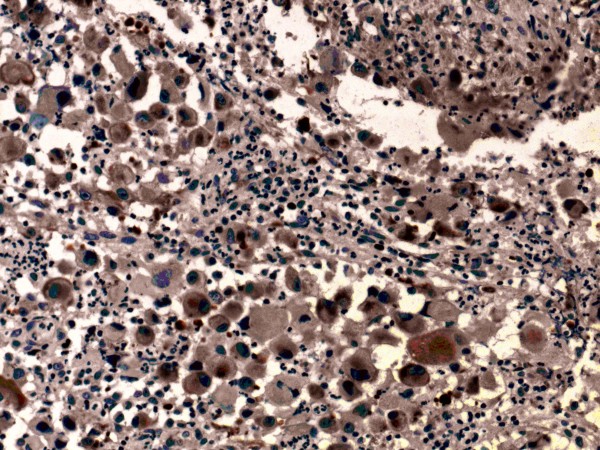
LCS tumor cells expressed Langerin (400×).

The LCS tumor cells expressed Langerin (Monoclonal mouse IgG (12D6), 1:200, Abcam. Figure 3), S-100 protein (Monoclonal mouse IgG (8B10), Abcam, 1:800, Figure 4), CD1a (Monoclonal mouse IgG (7A7), Abcam, 1:200, Figure 5), vimentin and CD68 (Monoclonal mouse IgG (3 F103), 1:50, Santa cruz) in the majority of cells. Lysozyme was weakly positive, and no tumor cells expressed CD20, CD3, CD30, AE1/AE3, LCA, CD23, CD35, CD56, ALK, CD138, PAX-5, CD15 and MPO. The Ki-67 proliferation index ranged from 70%-90%.

**Figure 4:**
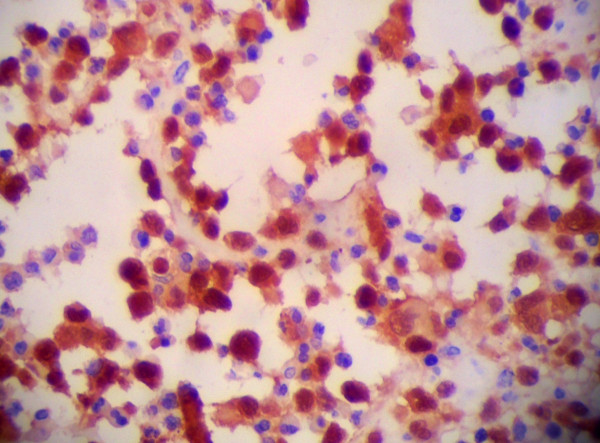
LCS tumor cells expressed S-100 protein (400×).

**Figure 5:**
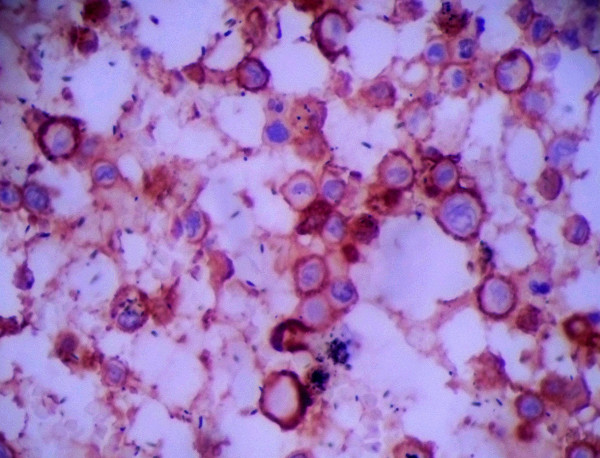
LCS tumor cells expressed CD1a (400×).

### Treatment and follow-up

After operation, a combination treatment protocol of cyclophosphamide, oncovin and prednisone (COP) was administrated. Six courses of cyclophosphamide (800 mg/m^2^, 1d), oncovin (2 mg/m^2^, 1d) and prednisone (60 mg/m^2^, 1-5d) were applied. At the same time, local radiation therapy with medical linear accerlerator (DT3600 cGy, 18 times) was carried out. But the result was negative, as the mass recurred at the right hip 2 months later. Another treatment protocols CHOP (cyclophosphamide, 750 mg/m^2^, 1d; doxorubicin, 50 mg/m^2^, 1d; oncovin, 1.4 mg/m^2^, 1d and prednisone, 100 mg/m^2^, 1-5d) was administrated with 6 courses. The patient died of metastasis in multiple organs (liver and lung) less than 1 year after being diagnosed with the disease.

## Conclusions

The first case of LCS was reported by Wood, who indicated that these tumor cells were antigen-presenting cells or dendritic cells that arose from hematopoietic or mesenchymal stem cells. The tumors of dendritic cells include Langerhans cell histiocytosis, Langerhans cell sarcoma, finger-like dendritic cell sarcoma/tumor, follicular dendritic cell sarcoma/tumor, and the declassified dendritic cell sarcoma, according to the 2008 WHO tumor classification [1]. LCS was defined as a tumor of Langerhans cell hyperplasia with markedly malignant cytological features. It can be considered to be a high-level variant of LCH at the beginning stages of LCS or as having progressed from LCH. The published literature has indicated that the majority of LCS cases are primary tumors and that only one reported case was a tumor that progressed from LCH [2].

The ages of reported patients of LCS ranged from 2 to 81 years, with a broad age distribution in adults and only 4 cases were younger than 18 years of age [3-5]. The ages of the five cases that were reported in the Chinese literature were 2, 18, 22, 41, 57 years (with a median age of 28 years). This age range is significantly younger than that of the cases reported in the English literature. Eighteen cases involved an organ (6 cases from the skin, 6 cases from the lymph nodes, 1 case from the lungs, 1 case from the mediastinum and 4 cases from bone and bone marrow) [6,7]. The majority of the cases of LCS involved skin, lymph node, bone and bone marrow, and most cases involved only a single organ. Bohn, et al. summarized 20 cases of LCS over 1992–2007 and found that most cases of LCS involved lymph node and skin and that only a small number of cases involved the lung, liver, spleen and bone marrow [8]. Lee, et al. summarized 19 cases of LCS from 1973–2006 and concluded that most of the patients had lymph node, spleen, liver, bone marrow, thymus, lung and kidney involvement, in addition to skin [2]. All of these reported literatures showed that one primary focus.

The diagnostic factors of LCS includes: typical histological features of tumor cells with characteristics of Langerhans cells; typical immunophenotype, such as, Langerin, CD1a, S-100; and ultra-structure characteristics, Birbeck granules within the cytoplasm. Occasionally, tumor cells with poorly differentiated or atypical morphology may lose the characteristics of ultra-structure or immunophenotype. Ben-Ezra J reported that only 3 cases presented Birbeck granules in the cytoplasm, among 9 cases of LCS [9]. Therefore, there are typical histological and immunophenotypic characteristics that can also be diagnosed as LCS. In our case, ultra-structural analysis did not indicate Birbeck granules in the cytoplasm of the neoplastic cells in this case.

The most effective treatment protocol for LCS is a combination of radiotherapy with chemotherapy. Some patients are treated with surgery and local radiotherapy, but the results are less positive. LCS has been reported to be completely restored after using MAID [10] or a modified ESHAP regimen (etoposide, carboplatin, cytarabine, and methylprednisolone) [11]. According to the literature, there is no relationship between the survival time, the number of organs involved, age and the treatment protocol. About 50% of patients have died within 1.5 years after diagnosis, so we conclude that LCS is a highly malignant tumor with a low survival rate and a poor prognosis. LCS may progress to leukemia [12], at which point, the immunophenotype of the tumor cells changes; it may be a LCS clinical manifestation to predict the progression. Tumor cells have also been shown to present a greater capacity for migration [13], and CD56 has marked prognostic significance and may prove useful as a clinically-relevant biological marker of Langerhans cell neoplasms [14]. However, no expression of CD56 was observed in the present case. A follow-up may have also been an effective means to identify the LCS in another report [7]. As for this case, chemotherapy and local radiation therapy were administrated after the operation. But the result was negative, as the mass recurred at the right hip 2 months later. One month after that, a chest X-ray showed a mass (3.9 cm × 2.9 cm) at the right lower lung. At the same time, the neoplasm recurred at the operation site. The treatment regime was so ineffective that the patient died of metastasis in multiple organs less than 1 year after being diagnosed with the disease.

In summary, we studied the clinical manifestation, immunophenotype and treatment course of a case of LCS with multiple primary foci. Due to its low incidence, it is vital to be able to recognize this highly malignant neoplasm and to differentiate it from other neoplasms. Accurate diagnosis is based on the morphologic features of the tumor cells and on the immunophenotype of the tumor cells.

### Consent

Written informed consent was obtained from the wife of the patient for publication of this Case Report and any accompanying images. A copy of the written consent is available for review by the Editor-in-Chief of this journal.

## Competing interests

All authors declare that they have no competing interests.

## Authors’ contributions

CSW was responsible for literature search and manuscript preparation. CFG and YPC participated in the discussion for histological diagnosis and manuscript preparation. JY collected the clinical data and postoperative clinical follow-up of the patient. CSW and HLi participated in the microscopic analyses. All authors read and approved the final manuscript.
